# Why an M1 Antagonist Could Be a More Selective Model for Memory Impairment than Scopolamine

**DOI:** 10.3389/fneur.2016.00167

**Published:** 2016-09-30

**Authors:** Arjan Blokland, Anke Sambeth, Jos Prickaerts, Wim J. Riedel

**Affiliations:** ^1^Maastricht University, Maastricht, Netherlands

**Keywords:** actelylcholine, Alzheimer, cognition enhancer, memory, deficit model

Since the early studies of Deutsch (1), the non-selective muscarinic receptor antagonist scopolamine has been used as a drug that impairs memory performance in man. The notion that scopolamine could be used as a pharmacological model of age-associated memory impairment and dementia further strengthened the cholinergic hypothesis of geriatric memory dysfunction by Bartus et al. (2). Since then, a vast amount of studies applied this model to induce memory impairments in young healthy subjects to model age-related memory disorders. At present, scopolamine is still considered to be the best model for inducing cognitive impairments in healthy subjects (3). Scopolamine is therefore used as a pharmacological model to test novel cognition-enhancing drugs in animals [e.g., Ref. (4–6)] and in humans [e.g., Ref. (7, 8)]. In clinical trials, scopolamine is in particular being used as a model for AD in which novel cognition-enhancing drugs are tested (see https://ClinicalTrials.gov).

Further efforts have been made to compare human and animal data with scopolamine to further validate the scopolamine as a model of cognitive impairments. For example, comparable tests were developed for humans and animals to allow cross-species comparison [e.g., Ref. (9, 10)]. Another effort is comparing scopolamine effects on brain imaging parameters (11, 12). These studies reveal great cross-species similarities in the effects of scopolamine in comparable cognitive tasks in humans and animals, and that the effects on central blood flow is also comparable. These studies have resulted in a huge database in which the effects of scopolamine on cognitive and non-cognitive functions have been documented. This also relates to doses and routes of administration. Furthermore, interactions with various other drugs (also non-cholinergic) have been documented, which supports the notion of drug interactions in memory functions. Taken together, scopolamine is considered as a golden standard for cholinergic deficits and the existing data were used as a reference for evaluating novel cognition-enhancing drugs.

Although scopolamine has this established (gold standard) status, there are also some important issues related with this drug. A first point is that scopolamine is binding to both peripheral and central muscarinic receptors [see Ref. (13)]. Thus, scopolamine binds to all five different muscarinic receptors which are located in the brain as well in the peripheral system. This may relate to the various side effects that can occur after administration of scopolamine. Typical side effects are: dry mouth or throat, dizziness, drowsiness, fatigue, nausea, light-headedness, and blurred vision [e.g., Ref. (9)]. A careful analytic review on the effects of scopolamine in animals has shown that scopolamine at low doses mainly affects attentional functions and that memory performance is only affected at higher doses [see Ref. (13)]. Moreover, at relative low doses, typical side effects (increased omissions and latencies in responding) can be observed in rodents that may have an impact on performance in memory tasks.

In humans, similar effects on sedation have been observed, but it has been suggested that these effects could be dissociated from the effects on memory impairments [see Ref. (14)]. Interestingly, these effects seem to be dependent on the route of administration. Thus, intramuscular or intravenous administration has shown robust effects on memory performance (3), accompanied with sedative effects. However, oral administration of scopolamine has resulted in sedative effects but in the absence of memory impairments (15, 16). Interestingly, the effects of intranasal scopolamine have also been investigated on side effects and cognitive performance (17, 18). This generally leads to a faster brain penetration and may have a stronger effect on brain function. Notably, no effects were found on cognition, and only some side effects were reported. However, it could be argued that the dose was too low (0.4 mg) or brain penetration was too fast in order to affect cognitive performance. Unfortunately, no plasma concentrations can be measured after intranasal administration, which makes it difficult comparing this with other routes of administration. Apparently, the effects of scopolamine on sedation are found in most clinical studies, whereas the effects on cognition are reported in fewer studies. Some experimental studies explicitly investigated the relation between sedation and cognition by comparing the effects of scopolamine and benzodiazepines (GABA_A_ agonist). In one study, the effects of scopolamine and lorazepam on cognition and sedation could not be separated (19). However, the effect of scopolamine and lorazepam can be separated on encoding processes, as shown in a repetitive priming paradigm (20). Another study also showed a differential effect of lorazepam and scopolamine on attention and working memory (21). Thus, the effects of benzodiazepines and scopolamine on arousal may be similar, but the effects on cognitive functions can be differentiated if specific tasks are used.

A subsequent study was able to show a one-sided dissociation between sedation and cognitive impairment (22). In this study, the effects of an H1 receptor antagonist (diphenhydramine) were compared with lorazepam and scopolamine. All drugs affected arousal but only scopolamine and lorazepam impaired memory. These findings were supported by the drug effects on EEG measures and indicate that the effects on arousal and memory were not interdependent. Although different studies suggest a differentiation between drug effects on arousal and memory (19, 22), a drug that only impairs cognitive functions and not the arousal state would be preferable. Moreover, this would show a double dissociation between arousal and memory performance (22).

Although the literature has shown robust effects of scopolamine on word learning (episodic memory task), some reported findings may suggest something else. Thus, scopolamine had a larger effect on immediate and delayed recall when presentation rate was fast (i.e., 1 word per 2 s), whereas it had only a marginal effect when presentation rate was 1 word per 5 s (23). The effects of scopolamine on word learning seem to be dependent on the pace of the task, which is related to the presentation time, inter-stimulus interval, and response-stimulus interval. These parameters are of key importance, whether attentional circuits in the brain are triggered. The faster the pace, the more declarative memory performance will become dependent on attentional constraints [or in essence, time constraints; see Ref. (24)]. Taken together, these findings suggest that the separation between arousal/attention and memory effects may not be easy to establish and require more variation of experimental parameters before this can be demonstrated.

As mentioned earlier, scopolamine is assumed to model the impaired cholinergic neurotransmission in AD. However, more recent studies have also shown other characteristic features of brain dysfunction in AD and the scopolamine model. For example, arterial spin labeled perfusion MRI studies have shown hypoperfusion mainly in the temporal lobe regions of AD patients (25, 26). In contrast, scopolamine has been found to mainly reduce cerebral blood flow in frontal areas (27–29). Although it may be questioned to what extend reduced blood flow in specific brain regions may relate to specific cognitive functions, these data do not support a strong face/predictive validity for scopolamine with respect to brain blood flow and the site of action in the brain. Although the above may caution the use of scopolamine as a model for memory impairment, scopolamine still is the golden standard for this purpose. The main advantage is that this drug is well characterized and there is enormous database to which the effects with new treatments can be compared with. For these reasons, it is obvious that scopolamine still will be used as a drug to induce memory impairments in animals and humans to model aging/AD-related memory dysfunctions.

Interestingly, a more specific cholinergic memory deficit model has been proposed based on selectivity of muscarinic receptors. Both the M1 and M2 receptors have been indicated as relevant for cognition, but most research has focused on the M1 receptor (30). It has been shown that the M1 receptor is more specifically located in cortical and hippocampal structures and that its expression in the body is limited [see Ref. (13)]. Moreover, the M1 receptor has been indicated to be related to cognitive deficits in AD (31, 32). Therefore, it has been suggested that blocking the M1 receptor could be regarded as a better model for age-associated and dementia-related memory deficits (13, 33). Biperiden, which is clinically used to reduce motor symptoms in Parkinson’s disease, is a relative selective muscarinic type 1 (M1) antagonist (34), and could be used as a drug to evaluate the effects on memory. Two human studies have shown selective effects of biperiden treatment on memory performance with only limited side effects (35, 36). A noteworthy feature of the postsynaptic M1 receptor is that an antagonist can impair memory performance and that an agonist can improve performance. Thus, the M1 receptor is also considered as a target to improve memory functions (37, 38). Various M1 agonists have been developed as drugs to improve memory performance in dementia and schizophrenia (39). One of the first (orthosteric) M1 agonist that showed efficacy in Alzheimer patients and schizophrenics was xanomeline (40, 41). However, this drug was not very selective for the M1 receptor and associated with dose-limiting side effects and was therefore not further developed. More recently, positive allosteric modulators (PAMs) have been developed which are more selective for the M1 receptor. One study in monkeys showed that an M1 positive allosteric modulator (PAM) improved the performance in an object detour test (42). Another study in humans showed that a PAM of the M1 receptor improved memory functions in a nicotine abstinence model (43). Taken together, the M1 receptor can be regarded as an interesting specific target for memory modulation.

The main mechanism by which M1 receptors can impair or improve memory is obviously via the cholinergic neurotransmission. However, additional mechanisms of action of M1 receptors have been described. For example, an *in vitro* study showed that blocking the M1 receptor decreases dendritic long-term potentiation (LTP) in the CA1 region of the hippocampus (44). Conversely, activation of the M1 receptors enhances LTP in the hippocampus (45). These effects are mediated by a co-localization of M1 and NMDA receptors and that activation of M1 receptors leads to enhanced NMDA receptor currents (46). This bidirectional modulation of LTP by M1 receptor modulation supports the notion that, aside from a cholinergic mechanism, LTP is also involved in the modulating the memory effects. It should be noted that the M1 receptor is also located in medium spiny neurons, where they are involved in neuronal plasticity and involved in motor functions (47). There are also studies showing that blocking the M1 receptors may affect more complex motor behavior [e.g., Ref. (48, 49)]. Actually, the M1 receptor antagonist biperiden was developed for this purpose. Although modulation of the M1 receptor in this structure may contribute to the behavioral effects of drugs that affect this receptor, it has been shown that the strongest effects of allosteric agonist were most pronounced in the hippocampus and to a lesser extend in the striatum (50). This may suggest that M1 drugs predominantly affect hippocampal-related functions. Along this line, the prescription of biperiden reports that amongst its side effects is memory loss. Moreover, this may further support the use of M1 antagonists as a model for selective memory impairment.

In summary, although scopolamine is being used to induce memory impairments in human subjects some aspects of this drug may caution the use of this drug to specifically impair memory performance. M1 antagonism can impair memory more specifically and M1 agonism (more specifically, PAMs) can improve memory. This strongly supports the notion that the M1 receptor is highly relevant and specific for memory. The use of M1 antagonist may offer a good alternative but more data are needed to support this claim.

## Author Contributions

AB wrote the paper. AS, JP, and WR commented on earlier versions of the manuscript.

## Conflict of Interest Statement

The authors declare that the research was conducted in the absence of any commercial or financial relationships that could be construed as a potential conflict of interest.
